# Research trends and hotspots in Adipose-Derived Stem Cell applications for peripheral nerve regeneration from 2000 to 2025: a bibliometrics visualization study

**DOI:** 10.1016/j.bas.2026.106124

**Published:** 2026-06-15

**Authors:** André S. Alves, Adriano Fabi, Daniel F. Kalbermatten, Srinivas Madduri

**Affiliations:** a Division of Plastic, Reconstructive and Aesthetic Surgery, Geneva University Hospitals, Geneva, 1205, Switzerland; b Bioengineering and Neuroregeneration Laboratory, Department of Surgery, University of Geneva, Geneva, 1205, Switzerland; c Department of Plastic, Reconstructive, Aesthetic and Hand Surgery, University Hospital of Basel, Spitalstrasse 21, Basel, 4031, Switzerland

**Keywords:** Adipose-derived stem cells, Peripheral nerve regeneration, Stem cell therapy, Tissue engineering, Nerve conduits, Autograft

## Abstract

**Introduction:**

Peripheral nerve injuries (PNIs) cause significant disability. Autologous nerve grafts remain the gold standard but are limited by donor-site morbidity, restricted graft availability, and inconsistent functional recovery. Adipose-derived stem cells (ADSCs) have emerged as promising adjuncts due to their accessibility and regenerative potential.

**Research question:**

How has global research on ADSCs in peripheral nerve repair evolved, and what are the leading contributors, dominant themes, and emerging trends?

**Material and methods:**

A bibliometric analysis was performed using the Web of Science Core Collection for studies published from January 2000 to September 2025. Predefined search terms for ADSCs and peripheral nerve repair were applied. VOSviewer (v1.6.20) and CiteSpace (v6.3.R1 Advanced) were used to build co-authorship, co-citation, and keyword co-occurrence networks and to assess thematic evolution.

**Results:**

The final dataset comprised 555 articles authored by 2750 researchers from 54 countries and 849 institutions, citing 16,466 references. Global output increased consistently after 2011, with a peak in 2019. When adjusted for population size, Switzerland showed the highest research intensity (3.22 publications per million inhabitants), followed by Sweden (1.81), Belgium (1.12), and Austria (1.11). Countries with the highest absolute output, including the United States (0.32) and China (0.12), demonstrated lower per capita contributions. Seminal work by Kingham and Coleman formed the conceptual basis for ADSC-mediated nerve regeneration. Keyword analysis revealed a shift from structural tissue engineering toward paracrine signaling, exosome-based strategies, and translational approaches.

**Discussion and conclusion:**

ADSCs are central to regenerative strategies for PNIs. Despite strong preclinical evidence, clinical translation remains limited, highlighting the need for standardized protocols and robust clinical trials.

## Highlights

• Research focus shifted from scaffold design to biological support of nerve regeneration.
• Paracrine signaling and secreted factors drive adipose stem cell nerve repair studies.
• Growing interest in exosomes as cell-free therapy for peripheral nerve injury.
• Keywords reveal transition from basic tissue engineering to translational research.

**Table:** Abbreviations

ADSCs	Adipose-Derived Stem Cells
BDNF	Brain-Derived Neurotrophic Factor
CNS	Central Nervous System
GDNF	Glial Cell Line-Derived Neurotrophic Factor
MeSH	Medical Subject Headings
NGCs	Nerve Guidance Conduits
NGF	Nerve Growth Factor
PNIs	Peripheral Nerve Injuries
PRP	Platelet-Rich Plasma
VEGF	Vascular Endothelial Growth Factor
WOSCC	Web of Science Core Collection

## 1 Introduction

Peripheral nerve injuries (PNIs) represent a significant clinical and socioeconomic burden worldwide (Bergmeister et al., 2020). They occur most frequently after trauma, including road traffic accidents, penetrating wounds, and iatrogenic damage during surgery. Epidemiological studies estimate an incidence ranging from 13 to 23 cases per 100,000 persons each year, with the highest prevalence in young and working-age individuals (Murphy et al., 2023; Zhang et al., 2022). The consequences of such injuries extend far beyond the immediate functional impairment: patients often face chronic pain, sensory disturbances, muscle weakness, and lifelong disability (Ciaramitaro et al., 2010). This long-term morbidity has a profound effect on quality of life and imposes a considerable financial cost, both in terms of direct healthcare expenses and indirect loss of productivity. In the United States, the average cost per patient in the upper limb has been estimated to exceed $47,000, with an average increase of $4623/year, illustrating the heavy economic burden associated with PNIs (Karsy et al., 2019).

The therapeutic options for repairing injured peripheral nerves remain limited. In contemporary practice, nerve transfers have emerged as a key strategy for selected injuries, particularly proximal lesions, as they allow reinnervation through shorter regeneration distances and may result in faster and more reliable functional recovery (Gutama et al., 2025). However, their indications remain specific and depend on the availability of suitable donor nerves, the timing of intervention, and the pattern of injury. When direct tension-free coaptation or nerve transfer is not feasible, the current gold standard remains the use of autologous nerve grafts, most commonly harvested from the sural nerve (Lans et al., 2023). Despite their widespread use, autografts are far from ideal. The procedure is constrained by the finite availability of donor nerves, requires additional surgical dissection, and inevitably causes donor-site morbidity such as sensory loss, neuroma formation, or chronic pain. Moreover, even with technically successful grafting, functional recovery is often incomplete, and results remain unpredictable, especially in long-gap injuries (Grinsell and Keating, 2014). These limitations have prompted an intensive search for alternative therapeutic strategies capable of improving outcomes while minimizing morbidity (Deumens et al., 2010).

Among the approaches that have gained prominence over the past two decades, nerve guidance conduits (NGCs) have emerged as an appealing substitute to autografts (Pfister et al., 2011). These tubular scaffolds, produced from natural or synthetic biomaterials, are designed to bridge nerve gaps by providing a permissive microenvironment that guides axonal extension. Several NGCs have entered clinical practice and shown encouraging results for short-gap repairs. Nevertheless, their effectiveness remains inferior to autografts in larger defects, largely because current conduits lack the biological activity and cellular support provided by the native Schwann cell niche (Carvalho et al., 2019).

To address this limitation, researchers have explored the integration of living cells into nerve conduits, aiming to replicate the biological guidance and trophic support normally exerted by Schwann cells. Because the direct clinical use of Schwann cells is restricted by harvesting difficulties and limited expansion potential, attention has shifted toward stem-cell–based strategies. Bone marrow–derived mesenchymal stem cells, induced pluripotent stem cells, and particularly adipose-derived stem cells (ADSCs) have been investigated as alternative cell sources (Zhang et al., 2020). Since Zuk et al. first isolated stem cells from human adipose tissue in 2001, ADSCs have attracted growing interest across regenerative medicine and surgery because of their accessibility, proliferative capacity, and multipotency (Zuk et al., 2001; Si et al., 2019). Compared with bone marrow–derived mesenchymal stem cells, ADSCs are more abundant and can be harvested through minimally invasive procedures such as liposuction, making them a practical and clinically appealing cell source (Zuk et al., 2003; LKanth et al., 2015; Gimble et al., 2007). In the context of peripheral nerve repair, their relevance lies not only in their ability to adopt Schwann cell–like phenotypes that support axonal regrowth and remyelination, but also in their strong paracrine activity, with secretion of neurotrophic and angiogenic mediators such as NGF, BDNF, GDNF, and VEGF that enhance the regenerative microenvironment (Cai et al., 2024; Lopatina et al., 2011; Marconi et al., 2012; Kingham et al., 2007; Chang et al., 2021). Their low immunogenicity and immunomodulatory properties further strengthen their translational potential, including possible allogeneic applications (Leto Barone et al., 2013). Together, these characteristics have placed ADSCs at the forefront of regenerative strategies in peripheral nerve surgery and make them particularly attractive for combination with bioengineered nerve conduits (Mohan et al., 2025).

The aim of the present work is to provide a comprehensive bibliometric analysis of the scientific literature on adipose-derived stem cells for peripheral nerve repair published between 2000 and 2025. By mapping the evolution of research activity, identifying geographic trends and collaborative networks, and examining the biological rationale underpinning ADSC use, this study seeks to highlight the growing role of ADSCs in the field of regenerative nerve surgery. Ultimately, this analysis intends to contextualize current evidence, outline emerging opportunities, and define priorities for future investigations.

## 2 Data and method

### 2.1 Search strategy

The search was conducted using the Web of Science Core Collection (WOSCC). The search strategy combined terms related to adipose-derived stem cells and peripheral nerve repair, applied to titles and abstracts as follows: ((TI=(adipose OR adipose-derived OR ADSC∗ OR ASC∗) OR AB=(adipose OR adipose-derived OR ADSC∗ OR ASC∗)) AND (TI=(nerve OR peripheral nerve OR nerve regeneration OR nerve repair) OR AB=(nerve OR peripheral nerve OR nerve regeneration OR nerve repair))) AND LA=(English) NOT TS=(central nervous system OR CNS OR spinal OR brain). Although Medical Subject Headings (MeSH) are not applicable to the Web of Science database, we designed a broad keyword-based strategy incorporating multiple synonyms to maximize sensitivity while maintaining specificity for peripheral nerve research. Importantly, inclusion and exclusion criteria were directly integrated into the search query (e.g., language restriction and exclusion of central nervous system–related studies), thereby limiting the need for extensive post hoc screening.

#### 2.1.1 Literature search

The search strategy covered publications from January 1, 2000, to September 5, 2025. After removing non-English papers and studies focused on the central nervous system, a total of 555 documents were retained for analysis. Some irrelevant articles, such as those primarily addressing anesthesiology-related nerve injuries, were also excluded during screening. For each included record, the following metadata were retrieved: title, authors, abstract, keywords, and cited references, all downloaded in plain text format. The final search was completed on September 5, 2025.

#### 2.1.2 Data extraction and bibliometric analysis

An initial overview of the dataset was obtained using the Analyze Results and Citation Report functions within the Web of Science platform. The bibliographic data were then processed with VOSviewer (version 1.6.20) and CiteSpace (version 6.3.R1 Advanced) to perform a more detailed bibliometric evaluation. The main steps included:

1. Exporting the full dataset, including author names, titles, sources, and abstracts. Data were formatted as plain text files for CiteSpace and as tab-delimited files for VOSviewer. Network analyses were carried out using different node types, including countries, institutions, authors, keywords, cited journals, cited authors, and cited references. The time span was set from 2000 to 2025, with one-year intervals. Each node on the resulting maps represented one element of the analysis (e.g., an author or institution).
2. Generating keyword co-occurrence maps and density visualizations in VOSviewer to highlight frequently used terms and illustrate their distribution across the field.
3. Applying CiteSpace to perform reference co-citation analysis, which allowed identification of landmark publications, influential research fronts, and emerging thematic trends.
4. Interpreting the visual outputs from VOSviewer and CiteSpace to characterize the structural and temporal evolution of research in ADSC-based peripheral nerve repair.

## 3 Results

**Fig. 1:**
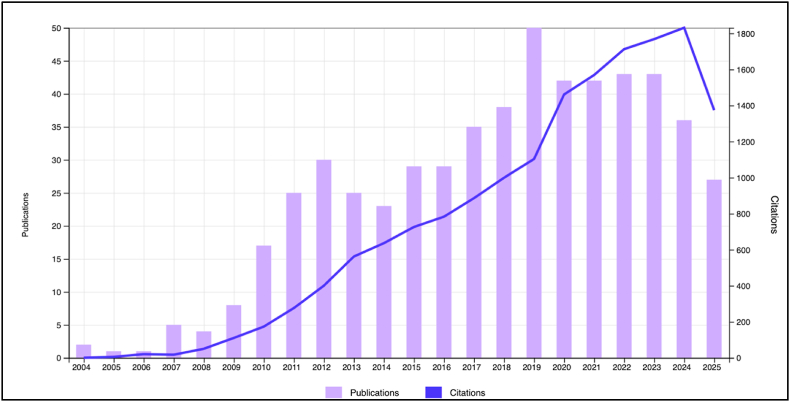
Annual distribution trend of publications and citations in the Web of Science database across years.

A total of 555 publications on adipose-derived stem cells (ADSCs) in peripheral nerve repair were retrieved from the Web of Science Core Collection. These studies involved contributions from 2750 authors, representing 54 countries and 849 institutions worldwide. The articles were published across 258 journals, collectively citing 16,466 references. On average, each article received 29.7 citations, with an annual citation rate of approximately 784 per year (Fig. 1).

### 3.1 Global publication trends

Analysis of publication output reveals the progressive growth of this research field over the last two decades. From 2000 to 2010, only a handful of papers were published each year, but beginning in 2011, the number of annual publications consistently exceeded twenty. Between 2011 and 2025, the mean annual output reached 22.2 papers, with several years surpassing this threshold. The most prolific year was 2019, during which 50 papers were published, marking the peak of scientific activity in this domain (Fig. 1). This pattern reflects a steady increase in global interest in ADSCs for peripheral nerve regeneration and the consolidation of the field into a recognized research hotspot.

### 3.2 Geographic distribution

**Fig. 2:**
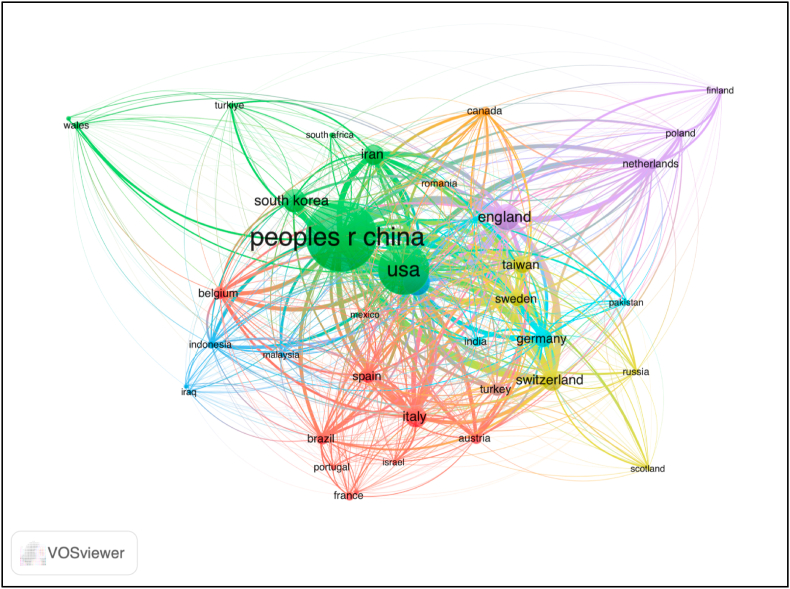
Network visualization of International collaborative analysis of studies related to using ADSC for the treatment of peripheral nerve repair and regeneration. Each cluster represents a group of country with common themes, where node size reflects country influence and link strength indicates the degree of collaboration or conceptual similarity.

The bibliometric analysis highlights the dominance of a few countries in driving this research area. China emerges as the leading contributor, accounting for 172 papers (31.9%), followed by the United States with 105 papers (18.9%). The United Kingdom ranks as the top European country with 39 publications (7.0%). Together, the top four publishing nations contribute 64.1% of the global output, demonstrating their central role in shaping advancements in ADSC-based nerve repair (Fig. 2). When adjusted for population size, the apparent dominance of high-output countries changes substantially. Switzerland shows the highest research intensity with 3.22 publications per million inhabitants (28 documents; population 8.7 million), followed by Sweden with 1.81 per million (19 documents; 10.5 million). Other high-performing countries include Belgium (1.12 per million; 13 documents; 11.6 million) and Austria (1.11 per million; 10 documents; 9 million). In contrast, countries with the largest absolute outputs demonstrate markedly lower per capita contributions. For example, China, despite producing the highest number of documents (172), has only 0.12 publications per million inhabitants (population 1410 million), while the United States shows 0.32 per million (105 documents; 331 million).Visualization through VOSviewer and CiteSpace confirms the close international collaborations underpinning this research. In particular, the United States exhibits strong academic connectivity with other countries, reinforcing its position as an influential hub within the global research network (Fig. 2).

### 3.3 Author productivity

The 555 publications involved 2750 authors, reflecting a highly collaborative field. The top 20 most prolific authors were associated with 237 publications; however, given the collaborative nature of the field, many of these articles are co-authored, and this figure does not represent unique contributions. (Table 1). Notably, Daniel F. Kalbermatten from the University of Geneva stands out as the most productive researcher, with 23 publications (4.1%). Other leading figures include Ching-Shwun Lin and Tom F. Lue from the University of California, San Francisco, each contributing 16 papers. Collectively, these authors, based in institutions across China, the United States, Switzerland, and the United Kingdom, have made significant contributions to advancing the understanding and clinical translation of ADSCs in nerve regeneration.

**Table 1:** The ranking of the top 20 authors in terms of the global publication output in the field.

Authors	Record Count	% of 555
Kalbermatten DF	23	4.144
Lin CS	16	2.883
Lue TF	16	2.883
Kingham PJ	15	2.703
Faroni A	14	2.523
Lin GT	14	2.523
Albersen M	13	2.342
Reid AJ	12	2.162
Terenghi G	12	2.162
Di Summa PG	11	1.982
Raffoul W	11	1.982
Fandel TM	10	1.802
Lee JY	10	1.802
Madduri S	9	1.622
Schaefer DJ	9	1.622
Shin AY	9	1.622
Wang Y	9	1.622
Kim SW	8	1.441
Peng J	8	1.441
Radtke C	8	1.441

### 3.4 Institutional contributions

**Fig. 3:**
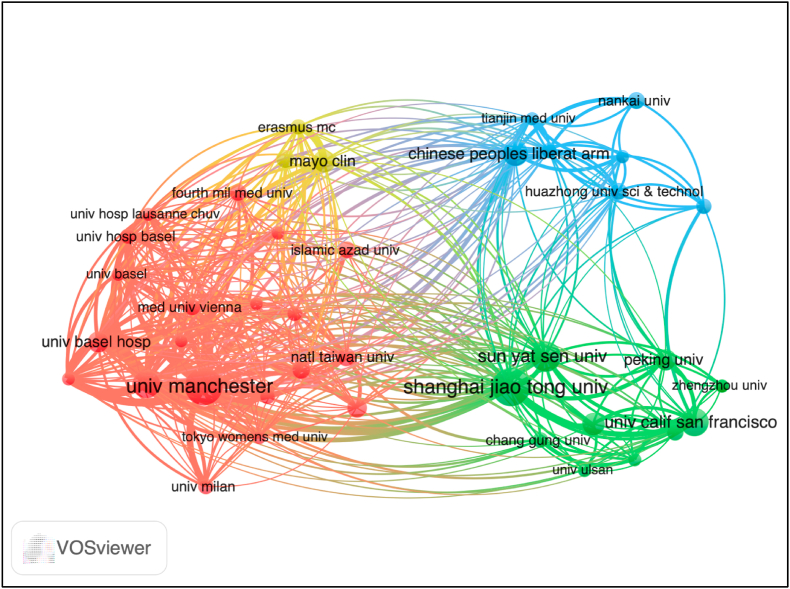
Network visualization of Institutions clusters based on shared references. Each cluster represents a group of Institutions with common themes, where node size reflects Institutions influence and link strength indicates the degree of collaboration or conceptual similarity.

**Fig. 4:**
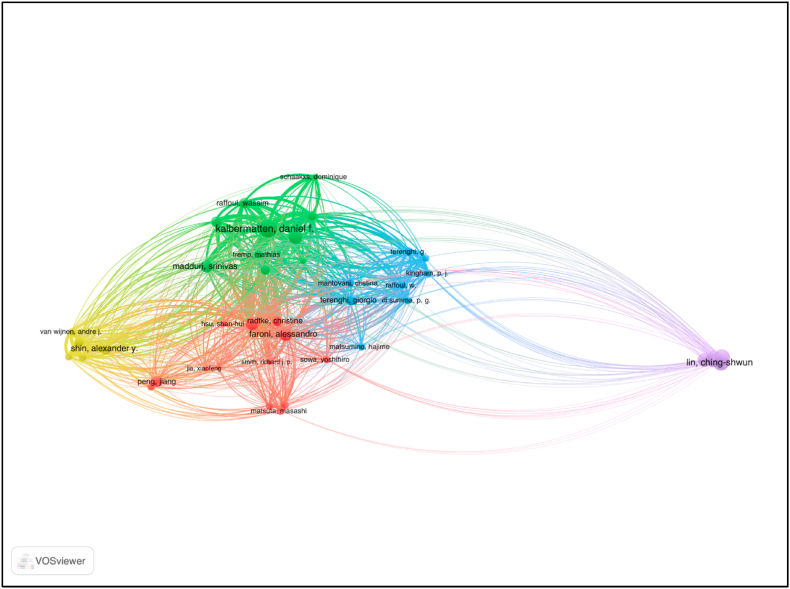
Network visualization of author clusters based on shared references. Each cluster represents a group of researchers with common themes, where node size reflects author influence and link strength indicates the degree of collaboration or conceptual similarity.

In terms of institutional productivity, a total of 849 organizations worldwide have been involved in this area of research. Fifteen institutions produced more than ten publications, reflecting the existence of concentrated centers of expertise. The Shanghai Jiao Tong University and the University of Manchester leads with 24 articles each, followed closely by Sun Yat Sen university, with 17 articles (Fig. 3). These results underscore the global nature of ADSC research, with strong representation across North America, Europe, and Asia. Co-authorship mapping further highlights the dense networks of collaboration, particularly within China and between Chinese institutions and international partners (Fig. 4).

### 3.5 Journal distribution

**Fig. 5:**
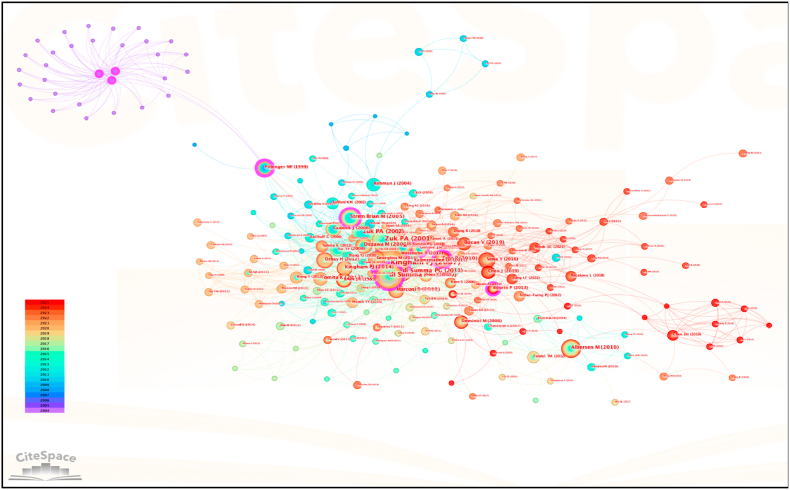
Bibliometric network visualization generated with Cites.Space. Reference co-occurrence network for the ADSC use nerve regeneration. Node colors correspond to the publication year, with the gradient indicating time (red denoting more recent works). Node size reflects the citation or co-citation frequency, with larger nodes representing highly influential references. Colored rings highlight citation bursts. Seminal studies, such as those by Kingham et al., Di Summa et al., and Zuk PA et al., appear as central and well-connected nodes, underscoring their pivotal role in this research field.

The literature on ADSC therapy for peripheral nerve regeneration is dispersed across 258 journals, yet a small subset dominates publication output. The most active journal is Tissue Engineering Part A (impact factor 2024: 2.9), which published 18 papers, followed closely by the Journal of Urology (IF 6.8) and Stem Cell Research & Therapy (IF 7.3), each with 17 papers. Visualization of journal output using VOSviewer indicates a temporal shift in preferred publishing outlets: earlier studies were concentrated in traditional tissue engineering journals, while more recent research has increasingly appeared in broader stem cell and regenerative medicine journals, reflecting the maturation and interdisciplinary expansion of the field (Fig. 5).

### 3.6 Cited references and citation patterns

The 555 articles collectively referenced 16,466 studies. Of these, 29 references had been cited more than 100 times and were therefore identified as the most influential works (Fig. 5) (Kingham et al., 2007; Coleman, 2006; Hsiao et al., 2012; di et al., 2010; Banas et al., 2008; Porzionato et al., 2018; Kim et al., 2013; Iohara et al., 2011; Albersen et al., 2010; Hong et al., 2019; Cai et al., 2009; McGonagle et al., 2017; Bucan et al., 2019; Georgiou et al., 2015; di Summa et al., 2011; Ding et al., 2015; Guo et al., 2016; Fandel et al., 2012; Qin et al., 2023; Castiglione et al., 2013; Liu et al., 2019; Erba et al., 2010; Hu et al., 2016; Carriel et al., 2013; Garcia et al., 2010; Chen et al., 2019; Oses et al., 2017; Ching et al., 2018; Yi et al., 2020)–(Kingham et al., 2007; Coleman, 2006; Hsiao et al., 2012; di et al., 2010; Banas et al., 2008; Porzionato et al., 2018; Kim et al., 2013; Iohara et al., 2011; Albersen et al., 2010; Hong et al., 2019; Cai et al., 2009; McGonagle et al., 2017; Bucan et al., 2019; Georgiou et al., 2015; di Summa et al., 2011; Ding et al., 2015; Guo et al., 2016; Fandel et al., 2012; Qin et al., 2023; Castiglione et al., 2013; Liu et al., 2019; Erba et al., 2010; Hu et al., 2016; Carriel et al., 2013; Garcia et al., 2010; Chen et al., 2019; Oses et al., 2017; Ching et al., 2018; Yi et al., 2020). Mapping of co-citation networks reveals clusters of foundational studies, with highly interconnected references underscoring their importance in shaping subsequent research directions.

### 3.7 Most cited publications

Citation analysis highlights several landmark papers that have profoundly influenced the field (Fig. 5). The most frequently cited work is Coleman's seminal 2006 article “Structural fat grafting: More than a permanent filler” published in Plastic and Reconstructive Surgery, which has accumulated 908 citations. The second most cited study, by Kingham et al. (2007) in Experimental Neurology, demonstrated that ADSCs can differentiate into Schwann-like cells and promote neurite outgrowth in vitro, receiving 502 citations. The third most cited article, published by Hsiao et al. in Stem Cells and Development (2012), compared the paracrine activity of stem cells derived from different tissues and has been cited 331 times. These highly influential publications illustrate both the translational relevance of ADSCs in reconstructive surgery and the mechanistic basis for their role in nerve regeneration.

### 3.8 Keywords analysis

Using VOSviewer, we identified 2047 keywords from publications on ADSC therapy for peripheral nerve regeneration. Of these, 196 keywords occurred at least five times. The most prominent term was “regeneration”, which appeared 123 times and showed the strongest association strength (882). This was followed by “differentiation” (112 occurrences, strength 805). Other frequently used terms included “tissue” (104 occurrences), “repair” (95 occurrences), and “transplantation” (90 occurrences), highlighting their central role in shaping the conceptual framework of this research field.

**Fig. 6:**
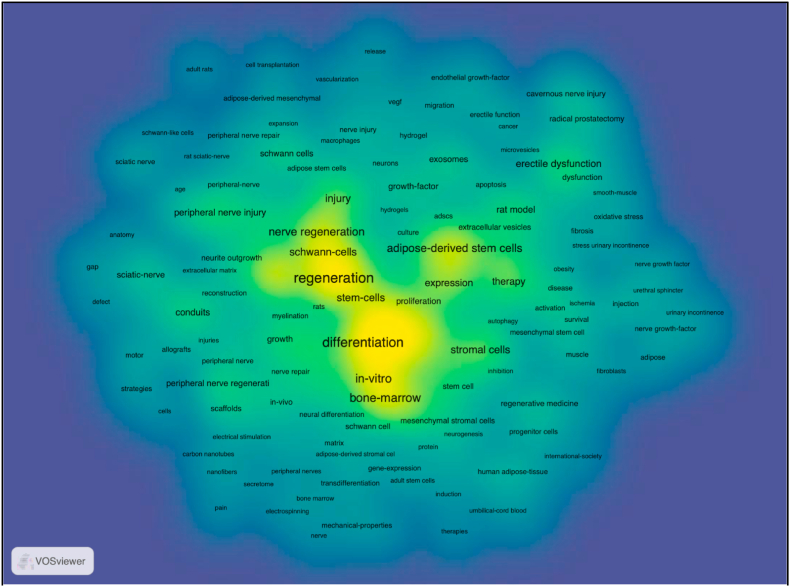
Keyword density maps were generated, where larger kernels represent terms with higher frequencies of occurrence.

**Fig. 7:**
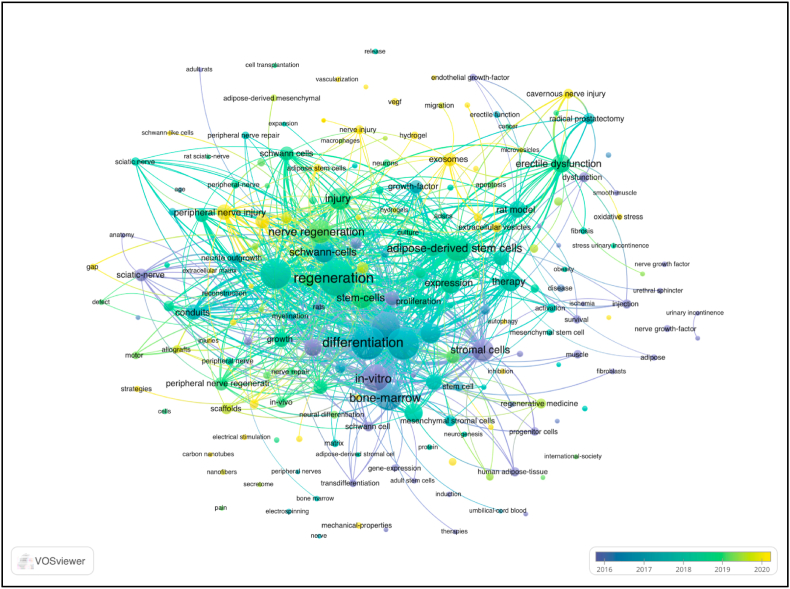
Keyword density maps were generated, where larger kernels represent terms with higher frequencies of occurrence.

Cluster analysis grouped the 186 high-frequency keywords into seven major clusters (Fig. 6). In this density maps visualization, each larger kernels represent terms with higher frequencies of occurrence. To further explore the temporal evolution of research themes, a keyword timeline was constructed which clearly depicts the dynamic shifts and emerging hotspots over time (Fig. 7).

### 3.9 Preclinical and clinical studies

To further characterize the translational stage of the field, publications were categorized according to experimental model. Most studies were preclinical, with 309 (55.7%) conducted in murine models, followed by a smaller number in rabbits (n = 12), porcine models (n = 7), and canine models (n = 2). In vitro studies accounted for 137 publications (24.7%), while only 3 studies (0.5%) involved human cadaveric models. In contrast, clinical research was limited and predominantly consisted of early-stage or indirect clinical evidence. Identified studies involving human applications included pilot clinical investigations, such as autologous adipose-derived stem cell (ADSC) transplantation within biodegradable nerve conduits (Klein et al.), as well as clinical procedures like lipofilling for fingertip reconstruction (Klein et al., 2016; Cerny et al., 2018). In addition, several publications focused on related clinical indications, particularly erectile dysfunction secondary to cavernous nerve injury, including network meta-analyses and narrative reviews integrating both animal and human data (Wani et al., 2022; Ghavam et al., 2025). A substantial proportion of the “clinical” literature consisted of reviews addressing the potential therapeutic role of ADSCs rather than reporting primary clinical outcomes. No large-scale randomized controlled trials or advanced-phase clinical studies were identified.

## 4 Discussion

The present analysis indicates that ADSCs research in peripheral nerve repair has progressively evolved from exploratory experimental work toward more translationally oriented regenerative strategies. A notable finding of this analysis is the concentration of publication output within a limited number of countries, with China representing the largest contributor in terms of volume. This observation reflects a strong research activity in the field of ADSC-based peripheral nerve regeneration, as captured by the bibliometric dataset. However, publication volume alone does not provide information regarding the translational impact or clinical applicability of these studies. The predominance of preclinical models within the dataset suggests that a significant proportion of research remains at an experimental stage, and therefore conclusions regarding clinical translation cannot be inferred from publication output alone. In contrast, co-authorship network analysis highlights the presence of structured collaborative clusters, particularly within European institutions, reflecting coordinated and multidisciplinary research efforts. Beyond publication counts, network-based analyses provide additional insight into the structural organization of the field. Co-citation mapping reveals that a limited number of seminal studies, particularly those by Kingham et al. and Zuk et al., occupy highly connected positions within the network, indicating their role as conceptual hubs linking experimental and translational research. Similarly, institutional collaboration networks demonstrate that certain centers, like University of Manchester, Shanghai Jiao Tong University, Umea University, Mayo Clinic, act as bridging nodes between otherwise distinct research clusters, reflecting not only productivity but also structural influence within the field. Keyword co-occurrence analysis further supports this observation, with terms such as “regeneration,” “Schwann cells,” and “transplantation” forming central nodes that integrate multiple research directions. Together, these findings indicate that the development of ADSC-based nerve regeneration is not only driven by publication volume but also by a relatively small number of highly influential studies and collaborative networks that shape the field's intellectual structure.

Taken together, these findings highlight a critical gap between biological validation and clinical implementation. While seminal studies such as Kingham et al. demonstrated that ADSCs can acquire Schwann-like phenotypes and significantly enhance neurite outgrowth in vitro, and di Summa et al. showed improved axonal regeneration in ADSC-seeded conduits comparable to Schwann cell constructs, these advances have not translated into widespread clinical adoption (Kingham et al., 2007; di Summa et al., 2011). The current dataset suggests that the vast majority of studies remain preclinical, with only a limited number progressing to early-phase clinical trials and an absence of large-scale randomized studies. In parallel, autologous nerve grafting continues to represent the clinical gold standard, underscoring the limited penetration of ADSC-based strategies into routine surgical practice. Notably, the persistent focus on Schwann-like differentiation, one of the dominant research themes, has not yielded scalable or standardized clinical solutions, likely due to technical complexity and regulatory constraints, suggesting a potential translational dead end. In contrast, the increasing prominence of keywords related to “nerve conduits,” “transplantation,” and “exosomes” suggests a shift toward biologically enhanced reconstructive strategies rather than purely structural approaches. Preclinical studies such as Klein et al. demonstrated that the use of autologous, undifferentiated ADSCs seeded within FDA-approved nerve conduits significantly improves motor and sensory nerve conduction velocity, supporting the feasibility of integrating ADSCs into existing surgical devices without the need for complex cell differentiation protocols (Klein et al., 2016). This approach is particularly relevant from a translational perspective, as it relies on readily available biomaterials and minimally manipulated autologous cells. In parallel, the growing focus on exosome-based therapies, reflected by the emergence of “exosomes” as a recent keyword cluster, highlights a transition toward cell-free strategies. Experimental work by Chen et al. showed that ADSC-derived exosomes enhance Schwann cell function, promote axonal regeneration, and improve myelination in vivo, suggesting a mechanism that may overcome key limitations of cell transplantation, including poor survival and regulatory constraints (Chen et al., 2019). Taken together, these converging bibliometric and experimental signals indicate that the most clinically relevant developments over the next 5–10 years are likely to involve (i) the integration of ADSCs into bioengineered nerve conduits using simplified, intraoperative protocols, and (ii) the emergence of cell-free, exosome-based therapies as scalable and potentially safer alternatives. In contrast, approaches requiring extensive in vitro manipulation, such as Schwann-like differentiation, appear less aligned with current translational trajectories despite their strong presence in the literature.

Despite these advances, important biological limitations remain, particularly regarding the persistence of transplanted ADSCs. Evidence suggests that transplanted cells decline rapidly over time, indicating that their therapeutic effects are primarily mediated through early paracrine signaling rather than long-term engraftment. (Skrypnyk, 2025). This observation provides a mechanistic basis for the evolving understanding of ADSC function, in which the regenerative benefit is driven less by structural integration and more by the transient secretion of bioactive factors capable of initiating sustained regenerative cascades. (Hong et al., 2019; Yang et al., 2024). Experimental studies have demonstrated that these vesicles can promote Schwann cell proliferation, enhance axonal regeneration, and improve myelination, supporting their potential as a cell-free therapeutic strategy. From a translational perspective, exosome-based approaches may offer advantages in terms of scalability, storage, and safety, although their clinical application remains at an early stage.At the molecular level, regulation of ADSC behavior by non-coding RNAs represents another emerging area of research. MicroRNAs such as miR-17 and miR-31 have been implicated in modulating proliferation, differentiation, and neurotrophic factor expression in ADSCs (Giammona et al., 2024; Li et al., 2013)While these findings provide important insights into the regulatory mechanisms underlying ADSC function, their translation into clinically applicable strategies remains limited, as most evidence is derived from in vitro or early preclinical studies.

Beyond molecular modulation, advances in cell culture technology are also being explored to preserve the stemness and enhance the therapeutic potency of ADSCs prior to transplantation. An emerging line of research has investigated how microgravity culture systems can enhance the therapeutic potential of ADSCs. Zhang et al. demonstrated that adipose-derived stem cells cultured in a microgravity bioreactor spontaneously aggregate into three-dimensional spheroids without the need for biomaterial scaffolds, thereby preserving their stemness and functional properties (Zhang et al., 2015). Compared with standard monolayer culture, spheroid-derived ADSCs showed increased expression of pluripotency markers, greater proliferative capacity, and improved colony-forming efficiency. Importantly, these cells retained enhanced multipotent differentiation potential and exhibited superior therapeutic efficacy in vivo, as evidenced by improved survival and functional rescue in a murine model of acute liver failure. These findings suggest that microgravity-induced spheroid formation may offer a powerful strategy to maintain ADSCs stemness and optimize their regenerative capacity. While this approach has yet to be translated into peripheral nerve repair, its potential to yield more potent and clinically effective ADSCs highlights a promising direction for future research in nerve tissue engineering.

A handful of early clinical applications, though limited in scope, have already suggested that ADSC-enriched biomaterials can improve motor and sensory recovery compared with conventional techniques (Prautsch et al., 2020). Strategies range from combining ADSCs with fibrin glue or hydrogels to seeding them within decellularized nerve scaffolds. A Phase I/II clinical trial (NCT02853942) investigated the use of autologous ADSCs for facial nerve paralysis, evaluating the safety and functional recovery following local injection into the damaged nerve region. Preliminary outcomes demonstrated no adverse events and suggested improvements in nerve conduction and muscle reinnervation, supporting the translational potential of ADSCs in cranial nerve repair. A complementary Phase I study (NCT04346680) is ongoing, exploring ADSCs' regenerative effects in peripheral facial nerve injuries, particularly assessing motor function recovery and synkinesis reduction. Beyond facial nerves, a clinical trial (NCT04654286) is evaluating ADSCs combined with a human amniotic membrane scaffold in brachial plexus injuries, aiming to determine whether the synergistic interaction between ADSCs' paracrine effects and the amniotic membrane's biocompatible structure can enhance axonal regeneration and functional recovery. Early reports indicate promising sensory improvement and electromyographic activity within the reinnervated muscles. While these reports are preliminary and frequently involve small, uncontrolled cohorts, they nevertheless underscore the translational potential of ADSCs therapy in reconstructive microsurgery.

Recent evidence also points to the growing relevance of allogeneic adipose-derived stem cells (AASCs) as a clinically viable option. A systematic review by Asch et al. (2025) analyzed over 950 patients across controlled clinical trials and confirmed that AASCs display a consistently favorable safety profile, with mostly mild adverse events and no evidence of malignant transformation or clinically significant allo-sensitization (Asch et al., 2025). Beyond their safety, AASCs demonstrated therapeutic potential in diverse conditions such as chronic wounds, Crohn's disease, osteoarthritis, and ischemic injury, largely through mechanisms of tissue regeneration, immune modulation, and vascular support. These findings suggest that allogeneic cell sources may overcome limitations associated with autologous harvesting, such as donor-site morbidity and variability in cell yield, while expanding accessibility for large-scale clinical use. Although phase III evidence is still lacking, this growing body of data reinforces the rationale for exploring AASCs not only in systemic diseases but also as a scalable adjunct in nerve tissue engineering strategies.

This study has several limitations that should be acknowledged. First, the bibliometric analysis was based exclusively on the Web of Science Core Collection database. Although this database is widely recognized for its reliability and structured indexing in bibliometric research, the use of a single database may have led to the omission of relevant studies indexed in other sources such as Scopus or PubMed. Consequently, some degree of selection bias cannot be excluded, particularly regarding regional publication patterns and journal coverage. Nevertheless, Web of Science remains one of the most commonly used databases for bibliometric analyses due to its high-quality metadata and citation tracking capabilities, and we believe that the overall trends identified in this study remain robust.

## 5 Conclusion

The use of adipose-derived stem cells, either alone or in combination with engineered nerve conduits, represents one of the most promising directions in modern regenerative medicine. While preclinical data strongly support their potential to enhance nerve regeneration, the translation into clinical practice will require a coordinated international effort emphasizing rigorous methodology, standardized protocols, and robust clinical trials. The complementary dynamics between China's high-volume research output and Europe's collaborative, high-quality translational research may play a decisive role in shaping the future of ADSC-based therapies. If these challenges can be addressed, ADSCs and their derivatives may eventually redefine the standard of care in peripheral nerve repair.

## Informed consent statement

Not applicable.

## IRB:

Not applicable.

## Author contributions

Conceptualization, A.S.A. and A.F.; methodology, A.S.A.; software, A.S.A.; validation, D.F.K. and S.M.; formal analysis, A.S.A.; investigation, A.S.A.; resources, A.S.A.; data curation, A.S.A.; writing—original draft preparation, A.S.A and A.F.; writing—review and editing, A.S.A., A.F., D.F.K and S.M.; visualization, A.S.A.; supervision, D.F.K. and S.M. All authors have read and agreed to the published version of the manuscript.

## Institutional review board statement

Not applicable.

## Data availability statement

All bibliometric data analyzed in this study are openly accessible through the Web of Science Core Collection.

## Funding

The Private Foundation of the Geneva University Hospitals and Swiss National Science Foundation.

## Declaration of competing interest

The authors declare the following financial interests/personal relationships which may be considered as potential competing interests:Srinivas Madduri reports financial support was provided by The Private Foundation of the Geneva University Hospitals and Swiss National Science Foundation. Srinivas Madduri reports a relationship with The Private Foundation of the Geneva University Hospitals and Swiss National Science Foundation that includes: funding grants. If there are other authors, they declare that they have no known competing financial interests or personal relationships that could have appeared to influence the work reported in this paper.
